# The relationship between distress tolerance and life satisfaction among young adults in Saudi Arabia

**DOI:** 10.3389/fpsyg.2024.1447466

**Published:** 2024-11-05

**Authors:** Hala Abd Ellatif Elsayed, Fatemah Aleriani

**Affiliations:** Clinical Psychology Program, Department of Health Sciences, College of Health and Rehabilitation Sciences, Princess Nourah Bint Abdulrahman University, Riyadh, Saudi Arabia

**Keywords:** distress, tolerance, life satisfaction, young adults, Saudi Arabia, wellbeing

## Abstract

**Purpose:**

This study examined the relationship between Distress Tolerance, defined as the individual’s ability to withstand psychological stress or endure negative emotions, and Life Satisfaction, the cognitive component of the individual’s subjective wellbeing, among Saudi young adults aged 20–30. The study aimed to understand the overall scores of distress tolerance, life satisfaction, and gender differences in these variables.

**Methods:**

Online questionnaires were distributed to 348 participants selected based on inclusion/exclusion criteria, using self-report scales: the Distress Tolerance Scale (DTS) and the Satisfaction with Life Scale (SWLS). The sample consisted of 77 males and 271 females, with a mean age of 1.8793 years (SD = 0.81931). Data were analyzed using the SPSS program.

**Results:**

The findings supported our hypotheses that (1) Saudi young adults have high overall scores of distress tolerance and life satisfaction, (2) there is a significant positive correlation between distress tolerance and life satisfaction, (3) there are no significant differences between males and females regarding overall distress tolerance and life satisfaction levels, although (4) a significant gender difference was found in the emotional regulation subscale of DTS. A linear regression analysis also showed that distress tolerance significantly predicts life satisfaction.

**Conclusion:**

Distress tolerance and life satisfaction are positively associated among Saudi young adults, with no significant gender differences in overall levels. However, differences in specific subscales, such as emotional regulation, warrant further investigation. These findings provide valuable insights for interventions to enhance this population’s wellbeing.

## Introduction

This study investigates the relationship between distress tolerance (DT) and life satisfaction among young adults in Saudi Arabia. By focusing on this unique cultural context, the research addresses a significant gap in the literature, as the influence of cultural, social, and economic factors on distress tolerance and life satisfaction still needs to be explored, particularly outside of Western settings.

Distress tolerance (DT) refers to an individual’s ability to endure psychological stress or negative emotions (Zvolensky et al., 2010). Previous studies have examined DT about various psychological and behavioral outcomes, such as psychopathology, personality traits, mood states, and coping behaviors (Simons et al., 2018; Anestis et al., 2012; Bernstein et al., 2011; Alaeddine, 2016; Felton et al., 2018; Ay and Aytas, 2018; Altairi, 2018; Houam and Zatout, 2018; Arici-Ozcan et al., 2019; Lass and Winer, 2020; Ghanbari et al., 2020). However, these studies predominantly focus on Western populations, leaving a gap in our understanding of how DT functions within different cultural environments, such as in Saudi Arabia. More recent research has underscored the importance of DT in managing stress and mitigating mental health issues (Del Giacco et al., 2023; Li et al., 2024).

Life satisfaction, a cognitive component of subjective wellbeing, reflects an individual’s overall evaluation of their quality of life. This evaluation process involves assessing life as a whole or specific life domains (e.g., family, work, social relationships) against personal standards and expectations (Mohamad et al., 2016). Various factors, including health, happiness, hope, psychopathology, substance abuse, and psychological distress, have been associated with life satisfaction (Rustoen et al., 2010; Bernstein et al., 2011; Aboalshamat et al., 2018; Altairi, 2018; Ghanbari et al., 2020). Recent studies have emphasized the role of cultural contexts in shaping these associations (Krys et al., 2023; Ji et al., 2022).

While regional studies have examined DT and life satisfaction individually, these investigations often incorporate other variables such as discrimination, personality characteristics, resilience, happiness, family income, social support, perceived stress, and depression (Altairi, 2018; Houam and Zatout, 2018; Aboalshamat et al., 2018; Khusaifan and El Keshky, 2016; Yildirim and Alanazi, 2018; Al-Juhani and Abu Asaad, 2019). However, the direct relationship between DT and life satisfaction in the context of young adults in Saudi Arabia still needs to be explored.

This study explores how distress tolerance influences life satisfaction among young adults in Saudi Arabia, considering the cultural and societal factors that may uniquely impact this relationship. It seeks to answer the following research questions: How do Saudi young adults score on distress tolerance and life satisfaction? Is there a significant relationship between an individual’s distress tolerance level and life satisfaction? Moreover, does this relationship differ between males and females? Finally, to what extent does distress tolerance impact life satisfaction in this population?

By addressing these questions, the study’s objectives include identifying the correlation between distress tolerance and life satisfaction among young adults in Saudi Arabia and exploring potential gender differences in these variables. The research also aims to raise awareness of how varying levels of distress tolerance may affect life satisfaction, guiding interventions to enhance wellbeing in this demographic.

The significance of this study lies in its potential to provide insights into the coping mechanisms of young Saudi adults and how their ability to tolerate distress impacts their overall life satisfaction. Understanding these dynamics is crucial, particularly in a rapidly changing society where young adults face multiple role transitions—such as leaving home, completing education, entering the workforce, and forming romantic partnerships—that can contribute to increased levels of distress. By comparing findings with those from Western populations, this research contributes to a broader understanding of cultural differences and similarities in the relationship between DT and life satisfaction.

The following sections will detail the study’s procedures, review related literature on DT and life satisfaction, describe the methodology, present the empirical results, and provide concluding remarks and recommendations based on the findings.

### Theoretical framework

This study is grounded in the Transactional Model of Stress and Coping, as proposed by Lazarus and Folkman (1984). This model provides a comprehensive lens to understand how individuals appraise and respond to stress, emphasizing the dynamic interaction between the individual and their environment.

The Transactional Model of Stress and Coping conceptualizes stress not as a static condition but as a process. This process involves an individual’s evaluation of their demands—stressors—and their perceived ability to cope with these demands. Central to this model are two critical types of appraisal: primary and secondary. In the primary appraisal phase, individuals assess whether an event or situation is irrelevant, benign-positive, or stressful. If the event is deemed stressful, it is further evaluated regarding its potential for harm or loss, threat level, or nature as a challenge (Lazarus and Folkman, 1984). This initial evaluation sets the stage for how the individual perceives and reacts to the stressor.

Following the primary appraisal, the secondary appraisal phase comes into play. In this phase, individuals assess their coping resources and options, determining their ability to manage, prevent, or adapt to the stressor. Effective coping in this context can lead to more favorable outcomes, such as reduced stress and enhanced wellbeing. In contrast, inadequate coping may result in heightened distress and adverse psychological outcomes (Folkman et al., 1986).

Distress tolerance plays a crucial role in both primary and secondary appraisals. This concept refers to an individual’s capacity to endure and withstand emotional distress. High levels of distress tolerance enable individuals to perceive stressors as less threatening, enhancing their ability to manage emotional responses effectively (Simons and Gaher, 2005). This capacity for tolerating distress is vital for achieving better psychological outcomes and higher levels of life satisfaction. Individuals with high distress tolerance are more likely to engage in adaptive coping strategies. These strategies include problem-focused approaches, such as problem-solving and seeking social support, and emotion-focused approaches, like positive reappraisal (Carver et al., 1989). By effectively managing distressing emotions, these individuals are better equipped to maintain positive emotional states, which, in turn, contribute to greater life satisfaction and overall wellbeing (Fredrickson, 2001).

The Transactional Model of Stress and Coping provides a robust theoretical framework for understanding the relationship between distress tolerance and life satisfaction. This framework underscores the importance of how stress is appraised and managed, highlighting the role of distress tolerance as a critical factor in achieving positive psychological outcomes.

## Literature review

Psychological distress is a prevalent mental health concern characterized by emotional suffering, often manifesting as symptoms of depression and anxiety (Arvidsdotter et al., 2015). Distress tolerance (DT) is the ability to withstand negative emotional and physiological states (Bernstein et al., 2011; Simons and Gaher, 2005). Numerous studies have explored the relationship between DT and psychological wellbeing, social interactions, and life transitions. However, the literature primarily reflects Western perspectives, with limited attention to the cultural nuances in non-Western settings, particularly in Saudi Arabia.

Recent research, such as the study by Lass and Winer (2020), has highlighted a negative correlation between DT and depressive symptoms, suggesting that individuals with lower DT are more prone to depression. This finding aligns with other studies demonstrating a moderate-to-large correlation between depressive symptoms and distress intolerance. These studies underscore the importance of DT as a potential risk factor for various psychopathologies, including risky behaviors such as substance use, non-suicidal self-injury, and bulimic symptoms (Felton et al., 2018; Anestis et al., 2007).

Further research has examined the interaction between DT and life events, particularly during adolescence, a critical developmental period for the onset of depression. Felton et al. (2018) found that adolescents with lower DT are more likely to develop depressive symptoms when exposed to adverse life events. This emphasizes the need for early intervention strategies that target DT to prevent the development of depression in vulnerable populations.

In addition to its role in depression, DT has been linked to eating behaviors and disorders. For instance, Anestis et al. (2007) found that individuals with low DT exhibit higher levels of urgency in predicting bulimic behaviors, suggesting that difficulties in tolerating distress contribute to the maintenance of bulimic symptoms. Similarly, Ay and Aytas (2018) observed that individuals with obsessive-compulsive disorder (OCD) had irregular eating habits and lower DT, highlighting the significant association between DT and eating behaviors.

Russell and Lincoln (2015) explored the impact of transitioning to parenthood on maternal wellbeing, finding that stressful stimuli, such as an infant’s crying, significantly decreased wellbeing and increased stress among new mothers. Their study underscores the role of DT in coping with the emotional challenges of parenthood, suggesting that higher DT is associated with better emotional regulation and maternal wellbeing.

The relationship between DT and cognitive factors has also been explored. Arici-Ozcan et al. (2019) examined the mediator role of mental flexibility and emotion regulation in the relationship between resilience and DT among college students. Their findings indicate that individuals with higher DT tend to have greater cognitive flexibility, experience less difficulty in emotion regulation, and exhibit increased resilience.

Therapeutic interventions have shown promise in improving DT. Ghanbari et al. (2020) compared the effectiveness of acceptance and commitment therapy (ACT) and quality of life improvement training (QOLT) on DT and self-destructive behaviors in substance abusers. Both methods significantly enhanced DT and reduced self-destructive behaviors, highlighting the potential of targeted interventions to improve DT in clinical populations.

While these studies offer valuable insights into the relationship between DT and various psychological outcomes, they are predominantly conducted in Western contexts, limiting their generalizability to non-Western populations. For example, Altairi (2018) investigated the relationship between discrimination, DT, and substance use among Arab American adults, finding that lower DT was associated with increased experiences of discrimination. However, this study did not explore how cultural factors specific to Arab communities influence these relationships.

In contrast, research focused on Saudi Arabia remains sparse. Al-Juhani and Abu Asaad (2019) studied the relationship between parental communication styles and life satisfaction among Saudi Arabian students, finding that effective parental communication is positively associated with life satisfaction. Aboalshamat et al. (2018) examined the relationship between resilience, happiness, and life satisfaction among medical and dental students in Saudi Arabia. Their results showed that higher resilience is associated with greater joy and life satisfaction, with gender and family income influencing resilience levels. However, these studies did not examine the role of DT, leaving a gap in understanding how DT interacts with cultural factors in shaping life satisfaction in this context.

Furthermore, a study by Khedr et al. (2023) explored the impact of resilience-based interventions on emotional regulation, grit, and life satisfaction among female Egyptian and Saudi nursing students. This randomized controlled trial demonstrated that resilience-based interventions significantly improved the participants’ emotional regulation, grit, and life satisfaction. This research highlights the importance of resilience in enhancing life satisfaction. However, it does not address the role of DT, underscoring the need for further investigation into how DT may intersect with resilience and cultural factors in Saudi Arabia.

Yildirim and Alanazi (2018) examined the mediation effect of stress in the relationship between gratitude and life satisfaction among Arabic-speaking students. Their findings suggested that higher levels of gratitude were associated with lower stress and greater life satisfaction. This study provides valuable insights into the role of gratitude and stress in enhancing life satisfaction among Arabic-speaking individuals, including those in Saudi Arabia. However, it does not explicitly address how cultural factors unique to Saudi Arabia, such as social norms and religious influences, might moderate these relationships. This gap highlights the need for further research that considers the distinct cultural context of Saudi Arabia in understanding how gratitude, stress, and life satisfaction interplay.

Hashemi et al. (2019) explored the mediating role of resilience in the relationship between cognitive emotion regulation strategies, distress tolerance (DT), and life satisfaction among addictive students. Their results indicated that positive cognitive emotion regulation strategies were directly and indirectly (mediated by resilience) correlated with life satisfaction. Distress tolerance was significantly related to life satisfaction among students exposed to addiction, with resilience serving as a mediator. While the study provides a comprehensive analysis of how resilience and DT influence life satisfaction in students at risk of addiction, it does not account for the cultural specifics of Saudi Arabia. In Saudi cultural contexts, family dynamics, societal expectations, and religious values could impact DT and resilience differently. Addressing these cultural nuances could enhance the understanding of how cognitive emotion regulation strategies and DT affect life satisfaction in Saudi Arabian students, thus filling a critical gap in the literature.

Moreover, studies such as those by Bernstein et al. (2011), Satici et al. (2020), and Son and Jeong (2023) have explored the impact of psychological distress on life satisfaction but have yet to consider mainly how cultural differences might moderate these effects. For instance, the impact of social norms, religious beliefs, and family dynamics on DT and life satisfaction in Saudi Arabia has yet to be thoroughly investigated.

### Critical evaluation and gaps in existing literature

While the existing literature offers a solid foundation for understanding the relationship between distress tolerance (DT) and life satisfaction, significant gaps still need to be addressed, particularly in non-Western contexts. The current body of research is primarily focused on Western populations, limiting the generalizability of these findings to Saudi Arabia. This study aims to fill this gap by investigating the relationship between DT and life satisfaction among young adults in Saudi Arabia, with a particular focus on cultural and societal influences unique to the region.

This study examines factors such as social norms, religious beliefs, and family dynamics to provide a more nuanced understanding of how DT and life satisfaction are interconnected within the Saudi Arabian cultural context.

Through this focused exploration, the research will contribute to the broader literature on psychological wellbeing in non-Western settings, offering culturally relevant insights applicable to Saudi Arabia.

Furthermore, the methodological approaches in existing studies warrant critical evaluation. Many studies employ cross-sectional designs, which, while helpful in identifying associations, limit the ability to draw causal inferences. This study acknowledges this limitation, as it is also cross-sectional. It aims to lay the groundwork for future longitudinal research that can more definitively explore the causal relationships between DT and life satisfaction in non-Western contexts.

Additionally, the literature often overlooks potential moderating variables such as gender, socio-economic status, and educational background. By incorporating these variables, this study aims to provide a more nuanced understanding of the factors influencing DT and life satisfaction among young adults in Saudi Arabia.

In summary, while the relationship between DT and life satisfaction has been well-documented, this study aims to contribute to the literature by focusing on a culturally specific context and addressing the gaps identified above. By doing so, it seeks to enhance our understanding of these constructs among young adults in Saudi Arabia and inform culturally relevant interventions to promote wellbeing.

#### Hypotheses

Thus, this study seeks to examine the following hypotheses:

1. Saudi young adults have high overall scores of Distress Tolerance and Life Satisfaction.
2. There is a significant relationship between Distress Tolerance and Life Satisfaction among Young Adults in Saudi Arabia.
3. There is a significant difference between males and females in Distress Tolerance and Life Satisfaction.
4. Distress tolerance affects life satisfaction among young adults in Saudi Arabia.

## Materials and methods

### Research design

The research philosophy is positivist and is designed based on a correlational research study to investigate whether there is an association between an individual’s level of distress tolerance and life satisfaction among young adults in Saudi Arabia. The Sociodemographic Information Sheet, Distress Tolerance Scale, and Satisfaction with Life Scale are distributed online via social media. Also, to see if distress tolerance and life satisfaction could be affected by gender differences and compare the two groups. The study may contain internal and external validity threats that should be considered. The translation of both scales might affect the internal validity threats, the Distress Tolerance Scale (Simons and Gaher, 2005), and the Satisfaction with Life Scale (Pavot and Diener, 1993). On the other hand, external validity can be influenced by the data collection procedure since the scales are administered online.

### Participants

The eligible participants of the study’s population are young Saudi adults aged 20–30. There is a relative scarcity of research focusing on the distress tolerance and life satisfaction of young adults in the Saudi Arabian context. Addressing this gap can contribute valuable insights to the existing literature and inform culturally relevant mental health practices. The study utilizes a sample size of 400 Saudi National Young Adult (20–30 years) participants based on Cochran’s Sample Size Formula. The calculation aims to determine a suitable sample size and estimate results with good precision for the whole population (Sarmah et al., 2013). The Cochran formula allows researchers to calculate an ideal sample size given a desired level of precision, desired confidence level, and the estimated proportion of the attribute present in the population. Cochran’s formula is considered especially appropriate in situations with large populations. The study sample was randomly selected based on the inclusion and exclusion criteria.

### Inclusion/exclusion criteria

This study focused on young adults, specifically Saudi nationals aged 20–30. The inclusion criteria were set to ensure a specific demographic relevant to the research questions. Adults who are not Saudi nationals and those under 20 or over 30 were excluded. This age range was selected due to these individuals’ transitional life stage, marked by increased independence, identity formation, and exposure to new social and professional pressures. These factors can significantly influence both distress tolerance and life satisfaction.

By including only Saudi nationals within this age range, the study aims to provide a more controlled examination of distress tolerance and life satisfaction within a relatively homogenous group experiencing similar cultural and societal contexts. However, this selection may also impact the study’s results, as it excludes non-Saudi nationals and older or younger individuals with different coping mechanisms and levels of life satisfaction.

### Impact of participant selection on study results

Including young adults in the specified age range provides insights into how distress tolerance and life satisfaction manifest during a critical developmental period. University students and professionals in this age group often face unique stressors, such as academic pressures, career decisions, and evolving social relationships. These stressors can significantly affect their distress tolerance and overall life satisfaction.

However, excluding non-Saudi nationals and individuals outside the 20–30 age range may limit the generalizability of the findings. Non-Saudi nationals in Saudi Arabia may experience different cultural and social dynamics that could influence their distress tolerance and life satisfaction differently. Similarly, individuals outside the specified age range may face other life challenges and stressors, affecting their coping mechanisms and wellbeing.

### Sampling process

Participants were recruited through social media platforms and university mailing lists to ensure a diverse sample. A random sampling technique was employed to mitigate selection bias and enhance the sample’s representativeness. The sociodemographic sheet included questions on gender, age, nationality, marital status, and educational level to explore their potential effects on the study variables.

### Measurements

#### Sociodemographic information sheet (Appendix C)

The sociodemographic sheet was self-developed for the study, and it consists of 5 questions: nationality, gender, age, marital status, and educational level. Nationality and age group help to identify the eligibility of the participants based on the inclusion\exclusion criteria. The gender item to help understand the gender differences between the participant’s responses, marital status, and educational level were selected to explore if they affected the study variables.

#### Distress Tolerance Scale (Appendix D)

The Distress Tolerance Scale (DTS) is a validated and reliable instrument developed by Simons and Gaher (2005) to measure an individual’s emotional distress tolerance. The scale comprises 15 items designed based on theoretical relevance and a review of related measures. It is divided into four subscales: perceived ability to tolerate emotional distress (e.g., “I cannot handle feeling distressed or upset”), subjective appraisal of distress (e.g., “My feelings of distress or being upset are not acceptable”), attention being absorbed by negative emotions (e.g., “When I feel distressed or upset, I cannot help but concentrate on how bad the distress feels”), and regulation efforts to alleviate distress (e.g., “When I feel distressed or upset I must do something about it immediately”). Participants rate these items on a 5-point Likert scale ranging from 1 (strongly agree) to 5 (strongly disagree). Item 6 is reverse scored. The total score ranges from 15 to 75, with higher scores indicating greater distress tolerance.

Simons and Gaher (2005) reported high internal consistency (*α* = 0.89) and adequate test–retest reliability (*r* = 0.61) for the DTS. The scale’s construct validity has been demonstrated through significant correlations with related constructs such as negative affectivity and experiential avoidance. Concurrent validity is supported by substantial associations with measures of anxiety sensitivity, indicating that individuals with lower distress tolerance are more likely to experience higher anxiety (Simons and Gaher, 2005).

The DTS was translated into Arabic using a back-translation method. The original English items were translated into Arabic by an expert proficient in both languages. Three bilingual Ph.D. holders in psychology independently evaluated the translation, and the final wording was agreed upon through consensus. The author obtained license and translation rights for the DTS.

#### Satisfaction with Life Scale (Appendix E)

The Satisfaction with Life Scale (SWLS), developed by Diener et al. (1985), consists of five items that assess an individual’s global cognitive judgments of their life satisfaction. Participants rate each item on a 7-point Likert scale ranging from 1 (strongly disagree) to 7 (strongly agree). The total score ranges from 5 to 35, with higher scores indicating greater life satisfaction.

The SWLS has demonstrated high internal consistency (Cronbach’s α = 0.87) and moderate temporal stability (test–retest reliability *r* = 0.82) (Pavot and Diener, 1993). Its construct validity is evidenced by significant correlations with other measures of subjective wellbeing and positive affect, distinguishing it from affective appraisals, which are more emotionally driven. Concurrent validity has been shown through its significant associations with mental health outcomes and quality of life indicators (Pavot and Diener, 2008).

For this study, the SWLS was translated into Arabic by an expert proficient in English and Arabic. Three bilingual Ph.D. holders in psychology reviewed the translation and agreed on the final wording. The original authors were permitted to use the SWLS for educational purposes.

### Methodology

#### Data collection procedure

First, the Institutional Review Board (IRB) approved the research, with approval number 21–0046 (Appendix A). The author obtained permission to use and translate the Distress Tolerance Scale (DTS) and the Satisfaction with Life Scale (SWLS) into Arabic through a back-translation process involving three bilingual Ph.D. holders (Appendix G).

Participants were selected using random cluster sampling to ensure a representative sample based on the inclusion and exclusion criteria. Before participation, all individuals were required to sign a written informed consent form (Appendix B), which provided detailed information about the study and assured confidentiality of their data. The questionnaire was administered online to reach a broader audience while considering potential biases introduced by this method, such as non-response bias and the digital divide. To mitigate these biases, reminders were sent, and the survey was designed to be accessible on various devices.

The sociodemographic information sheet included questions on gender, age, nationality, marital status, and educational level to explore whether these variables influenced the study outcomes.

#### Ethical considerations

All procedures adhered to the ethical standards of the institutional and national research committee. Informed consent was obtained from all participants, and their information was kept confidential. The study was conducted following the Declaration of Helsinki.

#### Data analysis

Data from the Sociodemographic Information Sheet (Appendix C), DTS (Appendix D), and SWLS (Appendix E) were exported to Excel and subsequently analyzed using SPSS for Windows, 64-bit edition, with a significance level set at 0.05.

Descriptive statistics, including mean and standard deviation, were calculated to provide an overview of the participants’ distress tolerance and life satisfaction levels. Pearson’s correlation was employed to examine the relationship between distress tolerance and life satisfaction based on prior research indicating significant associations between distress tolerance and psychopathology (Anestis et al., 2007; Felton et al., 2018; Ay and Aytas, 2018; Lass and Winer, 2020). Additionally, studies on life satisfaction (Bernstein et al., 2011; Hsieh et al., 2018; Satici et al., 2020) suggested a link between life satisfaction and mental health.

An independent samples *t*-test was conducted to determine if there were significant gender differences in distress tolerance and life satisfaction. Previous studies have shown that students with higher resilience report greater life satisfaction, and females often exhibit higher resilience levels (Aboalshamat et al., 2018).

Regression analysis was performed to assess the impact of distress tolerance on life satisfaction among young adults in Saudi Arabia. The DTS was scored based on the mean of its four subscales, with higher scores indicating greater distress tolerance. The SWLS scores were categorized into five satisfaction levels.

#### Limitations and bias mitigation

Potential biases in online data collection, such as non-response and selection biases, were addressed by sending reminders and ensuring the survey’s accessibility across various devices. Additionally, the random cluster sampling method helped achieve a representative sample, enhancing the generalizability of the findings.

#### Operational definition

Distress tolerance (DT) is the individual’s ability to withstand psychological stress or negative emotions (Zvolensky et al., 2010). A self-report scale, such as the Distress Tolerance Scale, can be measured like a 5-point Likert scale. Higher scores represent high distress tolerance. Averaging the results yielded distress tolerance scores, with lower scores indicating lower levels of distress tolerance (Simons and Gaher, 2005).

Life satisfaction is a cognitive assessment process that helps individuals evaluate their overall spectrum of quality of life. It is the mental aspect of an individual’s subjective wellbeing and represents quality of life (Mohamad et al., 2016). The Satisfaction with Scale (SWLS) helps to measure the individual’s level of satisfaction with their lives. It has a cutoff score between 5 and 35, and it is categorized into seven groups (31–35 Delighted, 26–30 Satisfied, 21–25 Slightly satisfied, 20 Neutral, 15–19 Slightly dissatisfied, 10–14 Dissatisfied, 5–9 Extremely dissatisfied) (Pavot and Diener, 1993).

## Results

The participants’ responses represent 87% of the actual utilized sample size for the study, consisting of 77 males and 271 females aged between 20 and 30 years (see Table 1). SPSS 64-bit edition was used to analyze the collected data from the distributed scales (DTS; Simons and Gaher, 2005, SWLS; Pavot and Diener, 1993) using a 0.05 level of significance to investigate the levels of distress tolerance and life satisfaction among young adults in Saudi Arabia. The participants were categorized by age group, marital status, and educational level.

**Table 1:** Descriptive statistics of the utilized sample.

Variables		Frequency	Percent
Gender	Male	77	22.1
	Female	271	77.9
	Total	348	100.0
Age group	20–23 years	140	40.2
	24–26 years	110	31.6
	27–30 years	98	28.2
	Total	348	100.0
Marital status	Single	239	68.68
	Married	97	27.87
	Divorced	11	3.16
	Widowed	1	0.29
	Total	348	100.0
Educational level	High school	48	13.79
	Bachelor’s degree	264	75.86
	Master’s degree	27	7.76
	Other	9	2.59
	Total	348	100.0

### Descriptive statistics

The descriptive statistics provide an overview of the participants’ demographic characteristics (see Table 1). The gender distribution shows that 22.1% of the participants were male, and 77.9% were female. Regarding age, 40.2% of the participants were between 20 and 23 years old, 31.6% were 24–26, and 28.2% were 27–30. The marital status data revealed that 68.68% of the participants were single, 27.87% were married, 3.16% were divorced, and 0.29% were widowed. Regarding educational level, 13.79% had a high school certificate, 75.86% held a bachelor’s degree, 7.76% had a master’s degree, and 2.59% indicated other educational qualifications.

### Hypothesis testing

*H*1: Levels of distress tolerance and life satisfaction.

The analysis of the overall scores of distress tolerance and life satisfaction is presented in Table 2. The mean score for the Distress Tolerance Scale (DTS) was 42.4914, with a standard deviation of 10.15684, indicating a relatively high level of distress tolerance among the participants. The Satisfaction with Life Scale (SWLS) had a mean score of 21.8534 with a standard deviation of 6.31168, classifying the participants as “slightly satisfied” with their lives.

**Table 2:** Overall scores of distress tolerance and life satisfaction.

	N	Range	Minimum	Maximum	Mean	SD
DTS total score	348	50.00	15.00	65.00	42.4914	10.15684
SWLS total score	348	30.00	5.00	35.00	21.8534	6.31168
Valid N (listwise)	348

*H*2: Relationship between distress tolerance and life satisfaction.

Pearson’s correlation examined the relationship between distress tolerance and life satisfaction (see Table 3). The results indicated a statistically significant positive correlation (*r* = 0.256, *p* < 0.001). This suggests that higher levels of distress tolerance are associated with higher levels of life satisfaction among young adults in Saudi Arabia.

**Table 3:** Pearson’s correlations of distress tolerance and life satisfaction.

		Distress tolerance total scores	Life satisfaction total scores
Distress tolerance total scores	Pearson’s correlation	1	0.256**
Life satisfaction total scores	Pearson’s correlation	0.256**	1

*n* = 348. **Correlation is significant at the 0.01 level (2-tailed). *p* < 0.001.

*H*3: Gender differences in distress tolerance and life satisfaction.

An independent-sample *t*-test examined gender differences in distress tolerance and life satisfaction (see Table 4). The results indicated no significant differences between males and females in distress tolerance (*p* = 0.334) and life satisfaction (*p* = 0.175). This suggests that gender does not significantly influence the levels of distress tolerance and life satisfaction in the sample.

**Table 4:** Independent-sample *t*-test gender differences on the distress tolerance scale and satisfaction with life scale.

		Levene’s test for equality of variances				*t*-test for equality of means				
		f	Sig.	t	df	Sig. (2-tailed)	Mean difference	Std. error difference	95% Confidence interval of the difference	
									Lower	Upper
Distress tolerance total scores	Equal variances assumed	0.022	0.881	0.968	346	0.334	1.27019	1.31177	−1.30986	3.85023
	Equal variances not assumed			0.971	122.993	0.334	1.27019	1.30838	−1.31967	3.86005
Life satisfaction total score	Equal variances assumed	0.036	0.849	1.358	346	0.175	1.10543	0.81410	−0.49577	2.70663
	Equal variances not assumed			1.378	125.187	0.171	1.10543	0.80218	−0.48215	2.69301

To Explore more in-depth the gender differences regarding the four dimensions subscales of the Distress Tolerance Scale. Tolerance, appraisal, absorption, and regulation: The statistical analysis results indicate no significant differences in distress tolerance, appraisal, and absorption Sig. (2-tailed) (Tolerance *p* = 0.973, Appraisal *p* = 0.802, and Absorption *p* = 0.369) ( p>0.05) . The regulation subscale indicates a significant difference between males and females *p* = 0.037 ( p<0.05) (see Table 5).

**Table 5:** Independent-sample *t*-test of gender differences in distress tolerance subscales.

		Levene’s test for equality of variances				*t*-test for equality of means				
		*f*	Sig.	*t*	df	Sig. (2-tailed)	Mean Difference	Std. Error Difference	95% confidence interval of the difference	
									Lower	Upper
Tolerance subscale	Equal variances assumed	1.257	0.263	0.078	346	0.973	0.00960	0.12234	−0.23103	0.25023
	Equal variances not assumed			0.078	122.458	0.938	0.00960	0.12240	−0.23269	0.25190
Absorption subscale	Equal variances assumed	1.052	0.306	0.900	346	0.369	0.12276	0.13641	−0.14553	0.39106
	Equal variances not assumed			0.921	126.787	0.359	0.12276	0.13327	−0.14096	0.38648
Appraisal subscale	Equal variances assumed	0.580	0.447	0.251	346	0.802	0.02541	0.10140	−0.17402	0.22485
	Equal variances not assumed			0.254	124.641	0.800	0.02541	0.10021	−0.17292	0.22375
Regulation subscale	Equal variances assumed	19.428	<0.001	2.099	346	0.037	0.24020	0.11444	0.01512	0.46529
	Equal variances not assumed			1.814	102.721	0.073	0.24020	0.13243	−0.02245	0.50286

### Regression analysis

A linear regression analysis was conducted to examine the effect of distress tolerance on life satisfaction. The regression summary (see Tables 6, 7) shows that distress tolerance accounted for 6.5% of the variance in life satisfaction [*R*^2^ = 0.065, *F*(1, 346) = 24.205, *p* < 0.001]. This indicates that distress tolerance is a significant predictor of life satisfaction among young adults in Saudi Arabia.

**Table 6:** Regression summary.

Model	R	R square	Adjusted R square	Std. error of the estimation
1	0.256^a^	0.065^a^	0.063^a^	6.11066^b^

a Predictors: (constant), distress tolerance.

b Dependent variable: life satisfaction.

**Table 7:** ANOVA of distress tolerance and life satisfaction.

Model		Sum of squares	df	Mean square	F	Sig.
1	Regression	903.820^a^	1	903.820^a^	24.205	<0.001
	Residual	12919.706^b^	346	37.340		
	Total	13823.526	347			

a Dependent variable: life satisfaction.

b Predictors: (constant), distress tolerance.

**Figure 1:**
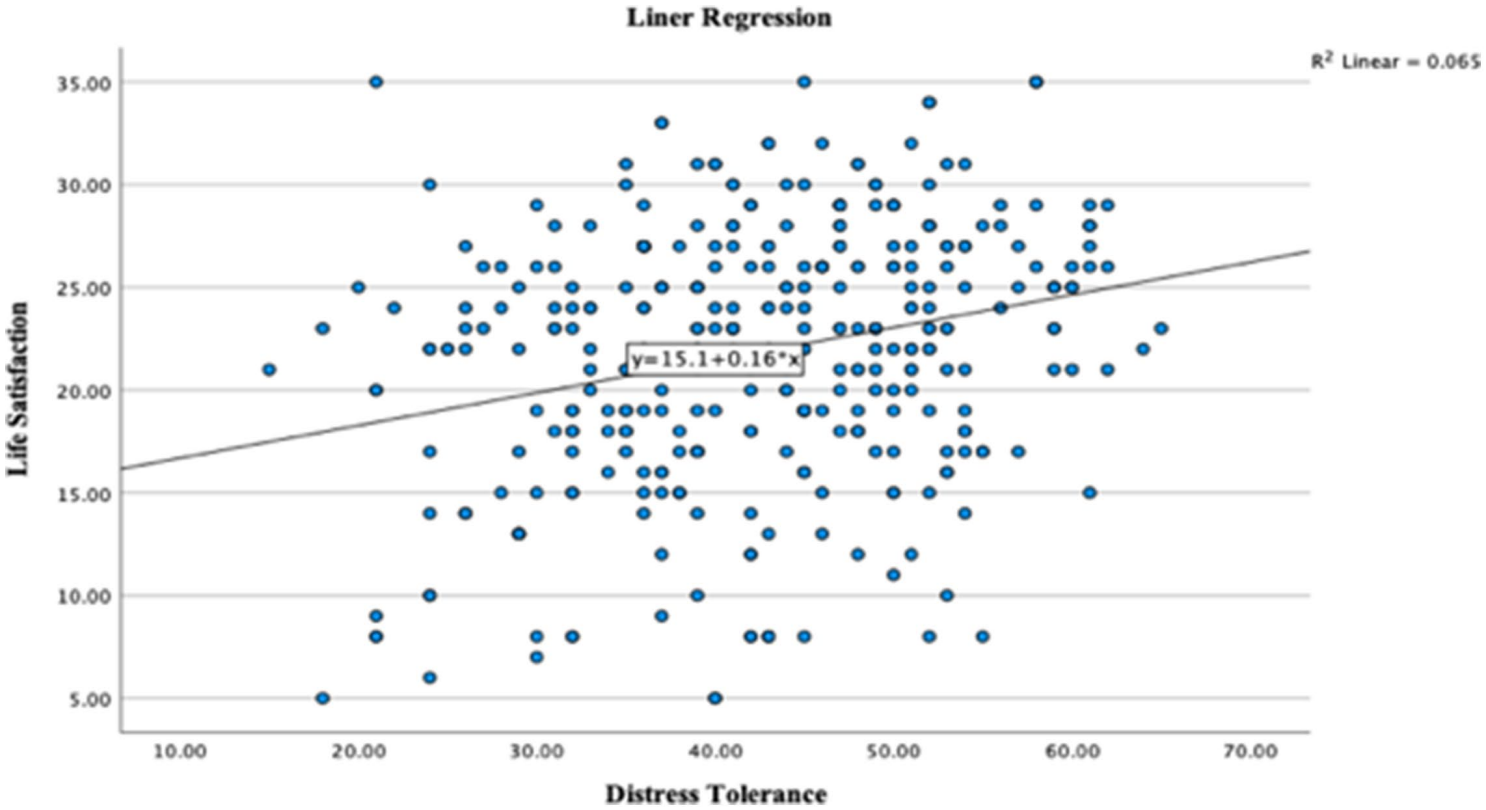
Scatterplot showing a linear relationship between distress tolerance and life satisfaction among Saudi young adults.

**Figure 2:**
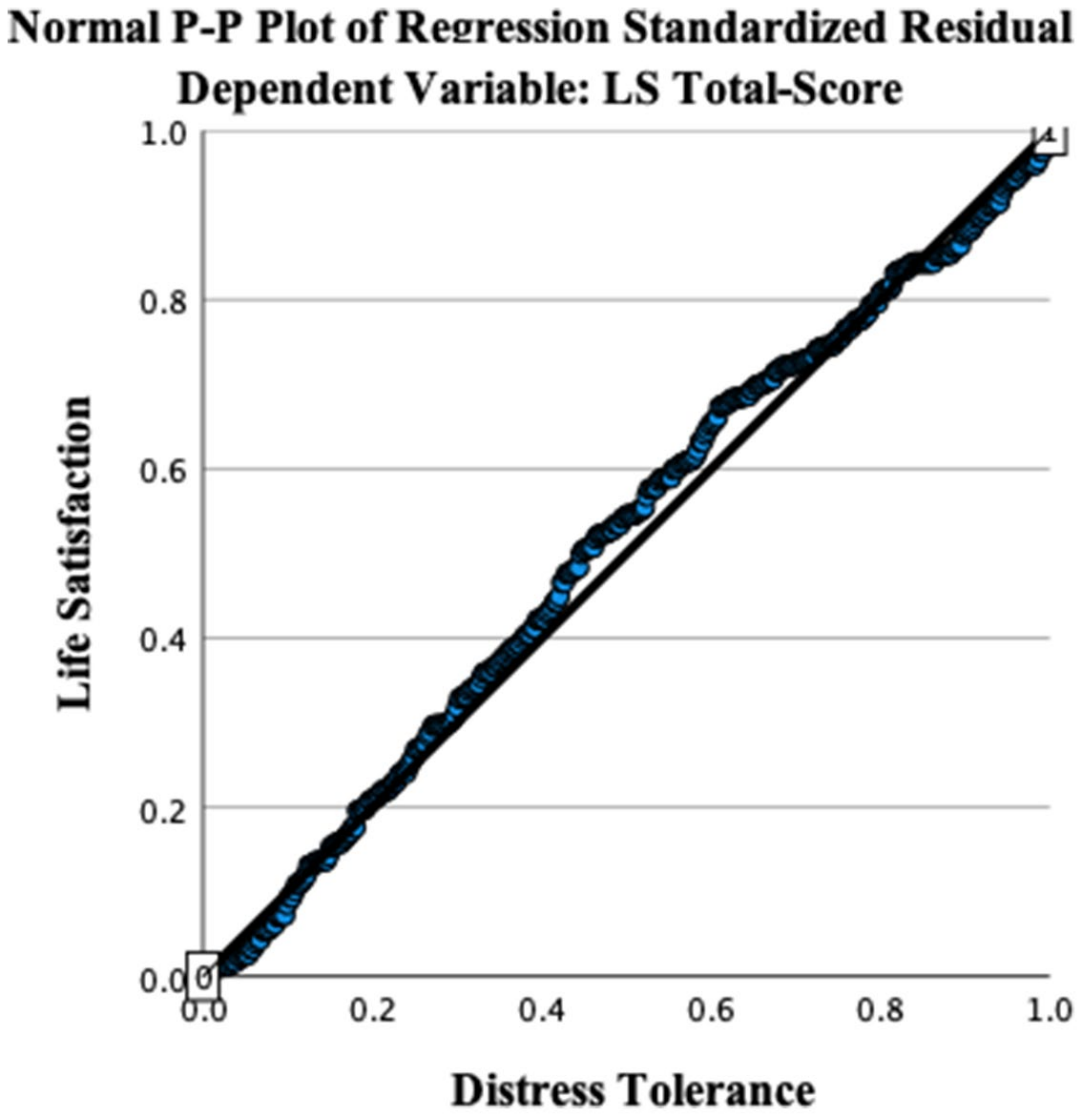
P–P plot shows the relationship between distress tolerance and life satisfaction levels among young Saudi adults.

Figures 1, 2 illustrate the linear relationship between distress tolerance and life satisfaction through a scatterplot and P–P plot.

### Reliability analysis

The reliability of the Distress Tolerance Scale (DTS) and the Satisfaction with Life Scale (SWLS) were evaluated using the Guttman split-half coefficient (see Table 8). The DTS demonstrated good internal consistency with a Guttman split-half coefficient of 0.726. The SWLS also showed good reliability with a Guttman split-half coefficient of 0.769 (see Table 9).

**Table 8:** Distress tolerance scale reliability.

Cronbach’s alpha	Part 1	Value	0.831
		N of items	2^a^
	Part 2	Value	0.409
		N of items	7^b^
	Total of items		15
Correlations between forms			0.690
Spearmen-brown coefficient		Equal length	0.817
		Unequal length	0.817
Guttman split-half coefficient			0.726

a Items numbers: 1, 2, 3, 4, 5, 6, 7, 8.

b Items numbers: 8, 9, 10, 11, 12, 13, 14, 15.

**Table 9:** Satisfaction with life scale reliability.

Cronbach’s alpha	Part 1	Value	0.769
		N of items	3^a^
	Part 2	Value	0.526
		N of items	2^b^
	Total of items		5
Correlations between forms			0.636
Spearmen-brown coefficient		Equal length	0.777
		Unequal length	0.783
Guttman split-half coefficient			0.769

a Items numbers: 1, 2, 3.

b Items numbers: 3, 4, 5.

### Summary of findings

In summary, the study found that young adults in Saudi Arabia exhibit high levels of distress tolerance and are slightly satisfied with their lives. A significant positive correlation exists between distress tolerance and life satisfaction, with distress tolerance significantly predicting life satisfaction. No significant gender differences were observed in distress tolerance and life satisfaction, except for the regulation subscale of distress tolerance, which showed significant differences between males and females.

## Discussion

This study examined the correlation between distress tolerance and life satisfaction among young adults in Saudi Arabia. It investigated whether there are gender differences in distress tolerance or life satisfaction levels and the effect of distress tolerance on life satisfaction. The findings revealed coefficient values indicating significant relationships between the variables (DT and LS).

First, the study suggests an ability to tolerate emotional distress and high overall life satisfaction scores among the targeted population. The study disclosed a positive correlation between distress tolerance and life satisfaction, consistent with previous findings (Anestis et al., 2007; Felton et al., 2018; Satici et al., 2020; Aboalshamat et al., 2018; Claydon et al., 2020; Lass and Winer, 2020). People who can tolerate and adapt to distressful emotions are more likely to be satisfied with their lives. Life satisfaction levels are positively associated with resilience, happiness, gratitude, and social support (Aboalshamat et al., 2018; Khusaifan and El Keshky, 2016; Yildirim and Alanazi, 2018; Al-Juhani and Abu Asaad, 2019) and negatively related to perceived stress and depression (Khusaifan and El Keshky, 2016; Yildirim and Alanazi, 2018). This explains the study’s findings, showing an association between distress tolerance and life satisfaction.

Secondly, while previous studies suggest gender differences in both DT and LS, this study found no significant differences between males and females. Previous research indicated that females have higher resiliency linked to higher life satisfaction (Aboalshamat et al., 2018), and men have higher distress tolerance (Simons and Gaher, 2005). However, the current study’s results could be due to the sample size and the disproportionate number of females (77.9%). Consistent with, no significant gender differences were found, possibly due to cultural factors unique to Saudi Arabia.

Thirdly, the study examined whether distress tolerance is associated with life satisfaction and whether there is a linear regression between them. The results indicate that distress tolerance level affects satisfaction with life. If individuals have difficulties tolerating distressful emotions, they may be less satisfied.

### Comparison with previous studies

Our findings are consistent with Son and Jeong (2023), who found a positive correlation between distress tolerance and life satisfaction. However, unlike, our study did not find significant gender differences, which may be attributed to cultural factors unique to Saudi Arabia. These discrepancies highlight the importance of considering cultural context when interpreting the relationship between distress tolerance and life satisfaction.

In summary, this study contributes to the existing literature by highlighting the positive correlation between distress tolerance and life satisfaction among young adults in Saudi Arabia. It underscores the need for future research to address the identified limitations and further explore the cultural nuances that may influence these relationships.

### Limitations

This study has several limitations that may affect the generalizability and interpretation of the results. First, the reliance on self-report measures introduces the possibility of bias, as participants may either overestimate or underestimate their distress tolerance and life satisfaction, and social desirability effects could further distort responses. Second, the sample’s gender imbalance, with a predominance of female participants, limits the ability to draw definitive conclusions about gender differences. Third, the cross-sectional design restricts the ability to infer causality between distress tolerance and life satisfaction. Finally, the cultural focus on Saudi Arabia’s young adults may limit the findings’ generalizability to other populations or cultural contexts. Future research should aim for a more balanced gender distribution, incorporate objective measures, employ longitudinal designs, and explore cross-cultural comparisons to strengthen the reliability and applicability of the findings.

## Conclusion

The study focused on exploring the level of emotional distress that individuals can tolerate and whether it interferes with the high standard of one’s life and satisfaction with life. Nowadays, changes are rapidly forming, which can cause high levels of distress among individuals, especially in the transition to young adulthood (Bonnie, 2015). Since previous studies investigating distress tolerance and life satisfaction in many different aspects like psychopathology, eating disorders, depression, and life transition, without discussing them together and examining if there is an association between them, this study is driven from this point. The study assumption formed that there is a relationship between distress tolerance and life satisfaction, gender differences regarding the proposed study problem, and if distress tolerance affects the life satisfaction levels among people. The study findings suggest a positive association between distress tolerance and life satisfaction among young adults without supporting gender differences. However, if the individual’s ability to tolerate distress and satisfaction with life decreases, the results of this research could aid in meeting the demands of the psychology industry, additionally, by growing people’s understanding of the effects of stressors and take this effect into account by providing training on strategies that can help increase the individual’s ability to tolerate distress. Tolerance to stressful situations will help a person’s ability to function more efficiently.

### Recommendations

Given the findings of this study, psychologists and mental health professionals must integrate distress tolerance training into their therapeutic practices. Mindfulness, cognitive-behavioral therapy (CBT), and stress management programs effectively enhance distress tolerance. By focusing on these methods, practitioners can help young adults improve their ability to manage negative emotions, increasing life satisfaction. Furthermore, although the study found no significant gender differences in distress tolerance, developing and implementing gender-specific approaches to emotional regulation remains essential. Tailoring interventions to address the unique needs of both males and females can ensure that psychological support services are equally beneficial across genders.

Educational institutions play a vital role in promoting emotional resilience among students. Based on the study’s findings, a strong case exists for developing and implementing programs that enhance distress tolerance within the educational curriculum. These programs could give students the tools to manage stress more effectively, improving their overall wellbeing. Public health campaigns should also be designed to raise awareness about the importance of distress tolerance and its significant impact on life satisfaction. By encouraging individuals to seek help and utilize available resources, these campaigns can contribute to the broader goal of improving emotional wellbeing within the community.

In light of the study’s limitations, future research should prioritize achieving a more balanced representation of male and female participants. This will allow a clearer understanding of potential gender differences in distress tolerance and life satisfaction. Additionally, researchers are encouraged to explore the role of distress tolerance as a mediator between psychopathology and life satisfaction. Such mediation analysis could provide deeper insights into how distress tolerance influences overall wellbeing. Moreover, it would be beneficial for future studies to include clinical populations, allowing for comparisons of distress tolerance levels between clinical and non-clinical groups, and understanding how distress tolerance functions across different age groups and psychological conditions could further refine intervention strategies. Finally, conducting longitudinal studies would be instrumental in establishing causal relationships between distress tolerance and life satisfaction. This approach would provide more robust evidence regarding the long-term effects of distress tolerance training and interventions.

This study contributes to the theoretical understanding of distress tolerance by highlighting its critical role in enhancing life satisfaction among young adults. The findings underscore the importance of distress tolerance as a significant factor in psychological wellbeing. Practically, the study suggests that interventions aimed at improving distress tolerance could have a substantial positive impact on life satisfaction. This insight calls for developing tailored psychological interventions and public health strategies to build emotional resilience and stress management skills. By addressing these recommendations, future research and practical applications can better support the wellbeing of young adults, thereby advancing the academic understanding and practical enhancement of distress tolerance and life satisfaction.

## Data Availability

The data that support the findings of this study are available upon reasonable request from the corresponding author.
