# Supplementary material for: The relationship between distress tolerance and life satisfaction among young adults in Saudi Arabia
Source: Front Psychol. 2024 Nov 5;15:1447466. doi: 10.3389/fpsyg.2024.1447466 (PMC11573513; doi:10.3389/fpsyg.2024.1447466)
Supplement: Supplementary file 1 [file Data_Sheet_1.pdf]

The Relationship between Distress Tolerance and Life Satisfaction among Young Adults in Saudi Arabia

Hala Alsayed[1], Fatemah Aleriani[2]

[1] *Associate Professor, Department of Health Sciences, Program of clinical psychology, Princess Nourah Bint Abdulrahman University, Riyadh, Saudi Arabia.*

[2] *Master Student, Department of Health Sciences, Program of clinical psychology, Princess Nourah Bint Abdulrahman University, Riyadh, Saudi Arabia.*

THE RELATIONSHIP BETWEEN DISTRESS TOLERANCE AND LIFE SATISFACTION AMONG YOUNG ADULTS IN SAUDI ARABIA.

Appendix A
IRB Approval

Kingdom of Saudi Arabia
Ministry of Education
Princess Nourah bint Abdulrahman University
(048)
Graduate Studies and Scientific Research Vice- Rectorate

المملكة العربية السعودية
وزارة التعليم
جامعة الأميرة نورة بنت عبدالرحمن
(٠٤٨)
وكالة الجامعة للدراسات العليا والبحث العلمي

IRB Registration Number with KACST, KSA: H-01-R-059

January 28, 2021
IRB Log Number: 21-0046
Project Title: The Relationship between Distress Tolerance and Life Satisfaction among Young Adults in Saudi Arabia
Category of Approval: EXEMPT

Dear Dr. Hala Abd Ellatif Elsayed and Fatemah S. Aleriani,

Thank you for submitting your proposal to the PNU Institutional Review Board. Your proposal was evaluated considering the national regulations that govern the protection of human subjects. The IRB has determined that your proposed project poses no more than minimal risk to the participants. Therefore, your proposal has been deemed ***EXEMPT*** from IRB review. Please note that this approval is from the research ethics perspective only. You will still need to get permission from the head of the department in PNU or an external institution to commence data collection.

Please note that the research must be conducted according to the proposal submitted to the PNU IRB. If changes to the approved protocol occur, a revised protocol must be reviewed and approved by the IRB before implementation. For ***any*** proposed changes in your research protocol, please submit a Request for Modification form to the PNU IRB. Please be aware that changes to the research protocol may prevent the research from qualifying for exempt review and require submission of a new IRB application or other materials to the PNU IRB. In addition, if an unexpected situation or adverse event happens during your investigation, please notify the PNU IRB as soon as possible. If notified, we will ask for a complete explanation of the event and your response.

Please be advised that regulations require that you submit a progress report on your research every 6 months. Please refer to the protocol number denoted above in all communication or correspondence related to your application and this approval. You are also required to submit any manuscript resulting from this research for approval by IRB before submission to journals for publication.

The researcher is personally liable for plagiarism and any violations of intellectual property rights.

For statistical services you are advised to contact the Data Clinic at the Health Sciences Research Center (hsrc-DC@pnu.edu.sa) or the Scientific Research Center at the Deanship of Scientific Research (dsr-rsc@pnu.edu.sa) extension 30711.

We wish you well as you proceed with the study. Should you have additional questions or require clarification of the contents of this letter, please contact me.

You can apply for research funding at (DSR-RS@pnu.edu.sa).

Sincerely Yours,

جامعة الأميرة نورة بنت عبدالرحمن
مجلس المراجعة المؤسسي
Institutional Review Board (IRB) 28 JAN 2021

Prof. Omar H. Kasule Sr.
Chairman, Institutional Review Board (IRB)
Princess Nourah bin Abdulrahman University, Riyadh, KSA
Tel: +966 548867916
E-mail: irb@pnu.edu.sa; ohkasule@pnu.edu.sa

الرقم: .... التاريخ: ..../..../....١٤هـ المشفوعات: ....

Appendix B
Informed Consent

FORM 10.05 **IRB Form**

Survey Cover Page / Consent

Effective Date: **PNU Institutional Review Board**

The Relationship between Distress Tolerance and Life Satisfaction among Young Adults in Saudi Arabia.

You are being asked to voluntarily participate in this survey research study. The purpose of the study is to understand the relationship between distress tolerance and life satisfaction. You are eligible to participate because you are part of the investigated population. We expect at least 400 will participate in the survey in Saudi Arabia.

If you agree to participate, your participation will involve completing a survey. It should take no more than 5-10 minutes. You may choose not to answer some or all of the questions. Your name will not appear on your completed survey, and no identifying information is being collected as part of this survey.

Any questions you have will be answered. You may leave the survey at any time before completing it. Whether you complete the survey or not will not affect your health care. There are no known risks from your participation. No direct benefit from your participation is expected. The information may help to complete the needed information for this study. There is no cost to you except for your time. You will not be paid for participation in this study.

Only the study team will have access to the information that you provide, which will remain anonymous. Data from all respondents will be summarized in reports.

You can obtain further information from the principal investigator, Fatemah Aleriani. If you have questions concerning your rights as a research subject, you may call the PNU Institutional Review Board office at 288-9999 ext. 26913.

Completing this survey indicates your voluntary agreement to participate. By participating in the survey, you are giving permission for the investigator to use your information for research purposes.

Thank you.

Principle investigator: Fatemah Aleriani
Masters student,
Fatimaer@live.com
Supervisor: Dr. Hala Alsayed
Associate Professor,
HAElsayed@pnu.edu.sa

Instructions:

- For the Distress Tolerance Scale (DTS) please think of times that you feel distressed or upset. Select the item from the menu that best describes your beliefs about feeling distressed or upset.
- For the Satisfaction with Life Scale (SWLS) indicate your agreement with each item by placing the appropriate number on the line preceding that item. Please be open and honest in your responding.
- It will take between 5-10 minutes.

Appendix C

Sociodemographic information Sheet

What is your nationality?

- Saudi
- Not Saudi

Please indicate your gander:

- Male
- Female

Please select the category that include your age:

- Under 20
- 20-23
- 24-26
- 27-30
- Above 30

What best describes your marital status?

- Single, not married.
- Married.
- Divorced.
- Widowed.

What is your educational level?

- High school.
- Bachelor.
- Masters.
- PhD
- Other

Appendix D

Distress Tolerance Scale

Directions: Think of times that you feel distressed or upset. Select the item from the menu that best describes your beliefs about feeling distressed or upset.

The statements	Strongly agree	Mildly agree	Agree and disagree equally	Mildly disagree	Strongly disagree
1. Feeling distressed or upset is unbearable to me.					
2. When I feel distressed or upset, all I can think about is how bad I feel.					
3. I can't handle feeling distressed or upset.					
4. My feelings of distress are so intense that they completely take over.					
5. There's nothing worse than feeling distressed or upset.					
6. I can tolerate being distressed or upset as well as most people.					
7. My feelings of distress or being upset are not acceptable.					
8. I'll do anything to avoid feeling distressed or upset.					
9. Other people seem to be able to tolerate feeling distressed or upset better than I can.					
10. Being distressed or upset is always a major ordeal for me.					
11. I am ashamed of myself when I feel distressed or upset.					
12. My feelings of distress or being upset scare me.					
13. I'll do anything to stop feeling distressed or upset.					
14. When I feel distressed or upset, I must do something about it immediately.					
15. When I feel distressed or upset, I cannot help but concentrate on how bad the distress actually feels.					

Appendix E

Satisfaction with Life Scale

Instructions: Below are five statements that you may agree or disagree with. Using the 1- 7 scale below, indicate your agreement with each item by placing the appropriate number on the line preceding that item. Please be open and honest in your responding.

- 7 - Strongly agree
- 6 - Agree
- 5 - Slightly agree
- 4 - Neither agree nor disagree
- 3 - Slightly disagree
- 2 - Disagree
- 1 - Strongly disagree

____ In most ways my life is close to my ideal.

____ The conditions of my life are excellent.

____ I am satisfied with my life.

____ So far, I have gotten the important things I want in life.

____ If I could live my life over, I would change almost nothing.

Appendix F

The Arabic Questionnaires

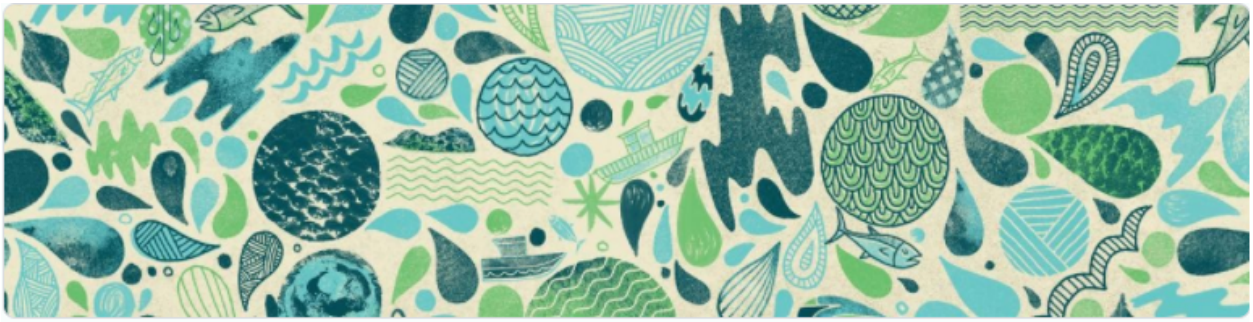

Section 1 of 4

العلاقة بين تحمل الضيق والرضا عن الحياة لدى الشباب في المملكة العربية السعودية.

موافقة مسبقة

تجري الباحثة دراسة بعنوان "العلاقة بين تحمل الضيق و الرضا عن الحياة لدى الشباب في المملكة العربية السعودية"، و ذلك لاكتساب فهم افضل بخصوص طبيعة العلاقة بين تحمل الضيق لدى الفرد و مستوى الرضا عن الحياة بين الشباب (٢٠ - ٣٠ عام) في البيئة السعودية. و منها لتزويد قاعدة بيانات تساهم في دعم البحوث اللاحقة. الوقت المستحق للإجابة على المقاييس (٥ - ١٠) دقائق، و لن يتم مشاركة اي من المعلومات التي تم تزويد الباحثة بها و ستبقى قيد السرية، و ذلك للحصول على درجة الماجستير في علم النفس العيادي بكلية الصحة و علوم التأهيل بجامعة الأميرة نورة بنت عبدالرحمن.

الباحث الرئيسي : فاطمة العرياني
إشراف : د. هالة السيد

هل ترغب في المشاركة في الدراسة؟ *

- نعم
- لا

المعلومات الديموغرافية

Description (optional)

الجنسية: *

- سعودي
- عير سعوي

الجنس: *

- ذكر
- أنثي

الفئة العمرية: *

- اقل من ٢٠ عام
- ٢٠ - ٢٣ عام
- ٢٤ - ٢٦ عام
- ٢٧ - ٣٠ عام
- اعلى من ٣٠ عام

الحالة الاجتماعية: *

- اعزب، غير متزوج
- متزوج
- مطلق
- أرمل

المستوى التعليمي: *

- ثانوي
- بكالوريوس
- ماجستير
- دكتوراه
- اخرى

مقياس تحمل الضيق

تعليمات الاختبار: يرجى التفكير في المرات التي تشعر فيها بالضيق او الاستياء واختر عبارة من القائمة تصف قناعاتك بأفضل طريقة تجاه الشعور بالضيق او الاستياء.

١. لا يمكن أن أتحمل الشعور بالضيق أو الاستياء. *

- اوافق بشدة
- اوافق
- لا اعرف
- لا اوافق
- لا اوافق بشدة

٢. عندما أشعر بالضيق أو الاستياء، كل ما يمكنني التفكير فيه هو مدى الاستياء الذي أشعر به. *

- اوافق بشدة
- اوافق
- لا اعرف
- لا اوافق
- لا اوافق بشدة

٣. لا أستطيع التعامل مع الشعور بالضيق أو الاستياء. *

- اوافق بشدة
- اوافق
- لا اعرف
- لا اوافق
- لا اوافق بشدة

٤. مشاعر الضيق شديدة جداً لدرجة انها تسيطر علي بشكل كامل. *

- اوافق بشدة
- اوافق
- لا اعرف
- لا اوافق
- لا اوافق بشدة

٥. لا يوجد شيء أسوأ من الشعور بالضيق أو الاستياء. *

- اوافق بشدة
- اوافق
- لا اعرف
- لا اوافق
- لا اوافق بشدة

٦. يمكنني تحمل الشعور بالضيق أو الاستياء كمعظم الناس. *

- اوافق بشدة
- اوافق
- لا اعرف
- لا اوافق
- لا اوافق بشدة

٧. مشاعري المتعلقة بالضيق أو الاستياء غير مقبولة لدي. *

- اوافق بشدة
- اوافق
- لا اعرف
- لا اوافق
- لا اوافق بشدة

٨. سأفعل أي شيء لتجنب الشعور بالضيق أو الاستياء. *

- اوافق بشدة
- اوافق
- لا اعرف
- لا اوافق
- لا اوافق بشدة

٩. يبدو أن الآخرين يستطيعون تحمل الشعور بالضيق أو الاستياء أفضل مني. *

- اوافق بشدة
- اوافق
- لا اعرف
- لا اوافق
- لا اوافق بشدة

١٠. شعوري بالضيق أو الاستياء هو دائماً أو عادةً محنة كبيرة بالنسبة لي. *

- اوافق بشدة
- اوافق
- لا اعرف
- لا اوافق
- لا اوافق بشدة

١١. أشعر بالخجل من نفسي عندما أشعر بالضيق أو الاستياء. *

- اوافق بشدة
- اوافق
- لا اعرف
- لا اوافق
- لا اوافق بشدة

١٢. تخيفني مشاعري بالضيق أو الاستياء. *

- اوافق بشدة
- اوافق
- لا اعرف
- لا اوافق
- لا اوافق بشدة

١٣. سأفعل أي شيء لأوقف الشعور بالضيق أو الاستياء. *

- اوافق بشدة
- اوافق
- لا اعرف
- لا اوافق
- لا اوافق بشدة

١٤. عندما أشعر بالضيق أو الاستياء، يجب أن أفعل شيئا حيال ذلك فوراً. *

- اوافق بشدة
- اوافق
- لا اعرف
- لا اوافق
- لا اوافق بشدة

١٥. عندما أشعر بالضيق أو الاستياء ، لا أستطيع سوى التركيز على سوء هذا الشعور في الواقع. *

- اوافق بشدة
- اوافق
- لا اعرف
- لا اوافق
- لا اوافق بشدة

مقياس الرضا عن الحياة

الإرشادات:

فيما يلي خمس عبارات قد توافق عليها أو لا توافق عليها. باستخدام المقياس من ١ إلى ٧ أدناه، حدد موافقتك على كل عنصر عن طريق وضع الرقم المناسب على السطر الذي يسبق هذا العنصر. من فضلك كن منفتح وصريح في اجاباتك.

٧ - أوافق بشدة
٦ - موافق
٥ - أوافق قليلاً
٤ - لا أوافق ولا أرفض
٣ - أعارض قليلاً
٢ - لا أوافق
١ - أرفض بشدة

1. في معظم الأحوال حياتي قريبة من الصورة المثالية. *

- ٧- أوافق بشدة
- ٦- موافق
- ٥- أوافق قليلاً
- ٤- لا أوافق ولا أرفض
- ٣- أعارض قليلاً
- ٢- لا أوافق
- ١- أرفض بشدة

٢. أحوال حياتي ممتازة. *

- ٧- أوافق بشدة
- ٦- موافق
- ٥- أوافق قليلاً
- ٤- لا أوافق ولا أرفض
- ٣- أعارض قليلاً
- ٢- لا أوافق
- ١- أرفض بشدة

٣. أنا راضٍ عن حياتي. *

- ٧- أوافق بشدة
- ٦- موافق
- ٥- أوافق قليلاً
- ٤- لا اوافق ولا ارفض
- ٣- أعارض قليلاً
- ٢- لا أوافق
- ١- أرفض بشدة

٤. حتى الآن، حصلت على الأشياء المهمة التي أريدها في الحياة. *

- ٧- أوافق بشدة
- ٦- موافق
- ٥- أوافق قليلاً
- ٤- لا أوافق ولا أرفض
- ٣- أعارض قليلاً
- ٢- لا أوافق
- ١- أرفض بشدة

٥. إذا تمكنت من عيش حياتي مجدداً، فلن أغير شيئًا تقريبًا. *

- ٧- أوافق بشدة
- ٦- موافق
- ٥- أوافق قليلاً
- ٤- لا أوافق ولا أرفض
- ٣- أعارض قليلاً
- ٢- لا أوافق
- ١- أرفض بشدة

Appendix G

The Approval of the Distress Tolerance Scale Use and Translation

Fatima December 4, 2020 at 1:55 AM F

Permission for the use of Distress Tolerance Scale

To: Jeffrey.Simons@usd.edu, jsimons@usd.edu

Hello,
Dear ,Jeffrey Simons

I hope this e-mail finds you will.

My name is Fatima, and I am a masters degree student at Princess Nourah Bin Abduallrahman University in Saudi Arabia.
The department of Clinical Psychology.

I'm working on my own research "The Correlation Between Distress Tolerance and Life Satisfaction among young adult between the age of 20-30 years in Saudi Arabia". I gone through the Distress Tolerance Scale (DTS) and it caught my attention. I'm looking forward to use it in my thesis.

I need your generous permission to use the DTS and translate it to Arabic language if there is no Arabic version to be suitable for my sample for academic propose.

Looking forward for your response.
Best regards,

Simons, Jeffrey S December 4, 2020 at 2:36 AM JS

Re: Permission for the use of Distress Tolerance Scale

To: Fatima

Siri found new contact info Jeffrey S Simons jeffrey.simons@usd.edu add...

Hello, you are welcome to use it. Attached is some relevant material.

--
Jeffrey S. Simons, PhD
Professor
Department of Psychology
The University of South Dakota
Vermillion, SD 57069
Phone: 605-658-3710
jsimons@usd.edu

See More from Fatima

Appendix H

Similarity Page

Thesis DT & LS

ORIGINALITY REPORT

0%	3%	3%	0%
SIMILARITY INDEX	INTERNET SOURCES	PUBLICATIONS	STUDENT PAPERS

PRIMARY SOURCES

Exclude quotes	On	Exclude matches	< 3%
Exclude bibliography	Off		
